# Improved production of melanin from *Aspergillus fumigatus* AFGRD105 by optimization of media factors

**DOI:** 10.1186/s13568-015-0161-0

**Published:** 2015-11-24

**Authors:** Nitya Meenakshi Raman, Pooja Harish Shah, Misha Mohan, Suganthi Ramasamy

**Affiliations:** Department of Biotechnology, Dr. G. R. Damodaran College of Science, 641 014 Coimbatore, India

**Keywords:** Melanin, Response-surface methodology, Plackett–Burman design, *Aspergillus fumigatus*

## Abstract

Melanins are indolic polymers produced by many genera included among plants, animals and microorganisms and targeted mainly for their wide range of applications in cosmetics, agriculture and medicine. An approach to analyse the cumulative effect of parameters for enhanced melanin production was carried out using response surface methodology. In this present study, optimization of media and process parameters for melanin production from *Aspergillus fumigatus* AFGRD105 (GenBank: JX041523; NFCCI accession number: 3826) was carried out by an initial univariate approach followed by statistical response surface methodology. The univariate approach was used to standardise the parameters that can be used for the 12-run Plackett–Burman design that is used for screening for critical parameters. Further optimization of parameters was analysed using Box–Behnken design. The optimum conditions observed were temperature, moisture and sodium dihydrogen phosphate concentration. The yield of every run of both designs were confirmed to be melanin by laboratory tests of analysis in the presence of acids, base and water. This is the first report confirming an increase in melanin production *A. fumigatus* AFGRD105 without the addition of costly additives.

## Introduction

Melanin has been reported to be produced by various bacteria (Lagunas-Munoz et al. 2006), fungi and many members of the plant kingdom. Biosynthesis of melanin in microorganisms has been largely associated with its prospects of being associated with UV protection, binding to antibiotics, resistance studies in pathogenic bacteria and its pathways have been targeted for potential targets for drugs in antimicrobial therapy. Wide uses of melanin have been previously reported in the field of cosmetics, protective agents in eye wear, as insecticidal crystals and photo protective creams (Zhang et al. 2007).

The production cost of any biotechnological process can be considerably reduced by optimization of the process (Sangkharak and Prasertsan 2007). The use of multivariate experimental design technique is becoming increasingly wide spread in applied biotechnology as it allows the simultaneous study of several control variables resulting in faster implementation and more cost effective approach than traditional univariate approaches (Nikel et al. 2005). The statistical method is a versatile technique for investigating multiple process variables because it makes the process easily optimized with fewer experimental trials (Bajaj et al. 2009).

Several experimental design models could be employed to reduce the number of experiments under different conditions. Plackett–Burman and Box–Behnken designs are among the most widely used statistical techniques for optimization of biological processes. The Plackett–Burman experimental design is a two-level factorial design, which identifies the critical physicochemical parameters by screening N variables in N + 1 experiments (Plackett and Burman 1946), but it does not consider the interaction effect among the variables. The variables that are found significant in this initial screening can be further optimized using response surface methodology (RSM) which is a collection of statistical techniques that uses design of experiments (DoE) for building models, evaluating the effects of factors and predicting optimum conditions (Abdel-Nabi et al. 1998). Now it is extensively applied in the optimization of medium composition, conditions of enzymatic hydrolysis, fermentation and food manufacturing processes. RSM which results from using Box–Behnken design was used for further study of the influences of major factors and interaction between them on the response value, which is based on the results of sole-experiment and Plackett–Burman (PB) design.

For this study, an initial standardization, optimizing the age of *A. fumigatus* AFGRD105 for maximum melanin production using standard media without any substitutions of other carbon or nitrogen sources was carried out. Application of statistical experimental methods to screen the significant medium components affecting melanin production and to evaluate the optimal levels of the significant variables has also been conducted by standardizing the parameters for study by an initial univariate approach followed by the use of statistical designs.

## Materials and Methods

### Microorganism

The identified strain *A. fumigatus* AFGRD105 (GenBank:JX041523) was grown on Sabourauds dextrose agar plates and slants at incubated for 5 days at 45 °C, followed which they were maintained at 4 °C for further subculturing. The entire set of experiments was performed using freshly subcultured strains. As major (wild-type) strains used in a study must be deposited in a publicly accessible culture collection the strain was deposited at National Facility for Culture Collection of Fungi (NFCCI -WDCM 932), Pune, India and the NFCCI accession number 3826 was obtained.

### Univariate approach confirming the optimum age, carbon and nitrogen source by submerged fermentation

*Age:* The strain was grown on Sabourauds Dextrose Agar (Dextrose 20 g, Peptone 10 g, Agar 15 g, Distilled Water 1000 mL) for a period of 10 days and checked for growth and melanization of the conidia present in the culture. Once the greying of the conidial samples was found, melanin isolation was repeated for each day till the tenth day. The lyophilized samples of melanin were weighed and plotted for comparison.

*Optimization of carbon sources*: *A. fumigatus* AFGRD105 was grown on medium substituted with various carbon sources (Dextrose, Galactose, Sucrose, Mannitol and Sorbitol) at the same concentration of Dextrose mentioned in the composition of the Sabourauds Dextrose Agar medium. Further, the optimum carbon source obtained was added in a range of 0.5, 1.0, 1.5, 2.0, 2.5, 3.0 g/100 mL and dry weight of the samples was measured at the optimized age of the culture and concentration of melanin was determined by isolating it from each sample.

*Optimization of nitrogen sources:* Medium with varying concentrations of Peptone and Yeast Extract (0.25, 0.5, 0.75, 1.0, 1.5 and 2.0 g/100 mL) was substituted in Sabourauds Dextrose Agar medium composition and the selected strain was inoculated for the optimized number of days. Dry weight of the fungus grown on each substituted media was measured. Melanin was isolated and the amount of melanin was determined.

*C:N ratio utilization:* For determining the C:N ratio, 250 mL shake flasks were used containing dextrose as the carbon source and peptone as the nitrogen source. Suspension of 5 × 10^6^ spores/ml were standardised in 500µL and inoculated into 100 mL of media and placed in a rotary shaker for a period of 10 days. Further addition of dextrose was carried out from the 6th day. In addition to dextrose and peptone, NaH_2_PO_4_, KH_2_PO_4_, MgSO_4_, CaCl_2_ and FeSO_4_ were added and pH was adjusted to seven prior to sterilization. The equivalent ratio of carbon and nitrogen with the amount of dextrose added is given in Table 1.

**Table 1:** Media composition for determination of C:N ratio

Initial concentration of dextrose	Peptone (g/L)	Initial C:N	Additional peptone added (g/L)	Equivalent C:N
4	2.66	7.2:1	16	25.25:1
8	2.66	16.2:1	12	25.25:1
16	2.66	20.65:1	4	25.25:1

### Multivariate approach

The optimum conditions for the age of the culture (time required for optimum production), carbon source and nitrogen source were subjected to other parameters using statistical package. The concentration of these sources was then subjected to high and low values averaging the optimum value. All the data were treated with the aid of design expert from Stat-Ease (8.0.7.1).

*Plackett Burman Experimental Design:* The Plackett–Burman (PB) design, an efficient technique for medium-component optimization (Yong et al. 2011), was used to pick factors that significantly influenced melanin production from *A. fumigatus* AFGRD105. PB design is one special type of a two-level fractional factorial design based on the incomplete equilibrium piece principle. It can pick up the main factors from a list of candidate factors with the least number of experiments. Total number of trials to be carried out according to the Plackett–Burman is n + 1, where n is number of variables (medium components). Each variable is represented at two levels, high and low, which are denoted by (+1) and (−1), respectively. Eleven process parameters including pH, temperature, inoculum volume, incubation time, substrate, moisture content, NaH_2_PO_4_, KH_2_PO_4_, MgSO_4_, CaCl_2_ and FeSO_4_ were added in two levels of +1 and −1. This design is used for the characterization of the model that results in the significant variable where there is no interaction among the factors (Plackett and Burman 1946). The statistical significance of this model was given by Fischer’s test and ANOVA. The lists of the factors involved in the experimental design are given in Table 1 and the design parameters are given in Table 2.

**Table 2:** Experimental variables at different levels used for production of Melanin

Factors	Variable	Units	Experimental values	
			Low (−1)	High (+1)
1	pH		5	9
2	Temperature	°C	40	50
3	Inoculum volume	CFU/Ml	0.5	1.5
4	Incubation time	Days	3	7
5	Substrate	g	3	7
6	Moisture content	%	10	25
7	Sodium dihydrogen phosphate	g	10	14
8	Potassium dihydrogen phosphate	g	1	3
9	Magnesium sulphate	g	0.1	0.5
10	Calcium chloride	g	0.5	1.0
11	Ferrous sulphate	g	0.1	1

*Optimization of growth parameters for Box Behnken design:* Independent positive variables obtained after PB design was optimized by Response surface methodology (Table 3). In general, response surface methodology contains Box–Behnken (BB) design and Central Composite Design (CCD). CCD is a five-level fractional factorial design which is tensely dependent on the accuracy of the central point. Based on the results of the PB design, the BB design was conducted to gain the optimal levels of the main factors picked out by PB experiment. Each variable was studied at three different levels of low, intermediate and high (−1, 0, +1). Experimental design included 17 flasks for the strain with three factors (Table 4). Response surface graphs were obtained to understand the effect of the variables, individually and in combination, and to determine their optimum levels for maximum melanin production. The data obtained from 17 experiments, were used to find out the optimum point of the process parameters by using Box–Behnken Design in Response surface methodology.

**Table 3:** Twelve trial Plackett Burman design matrix for the experimental variables with coded values for melanin production

Runs	Experimental parameter											Response (mg/L)
	1	2	3	4	5	6	7	8	9	10	11	
1	−1	−1	+1	−1	+1	+1	−1	+1	+1	+1	−1	15.6
2	−1	−1	−1	+1	−1	+1	+1	−1	+1	+1	+1	16.7
3	−1	+1	+1	−1	+1	+1	+1	−1	−1	−1	+1	17.5
4	+1	−1	+1	+1	+1	−1	−1	−1	+1	−1	+1	11.8
5	−1	−1	−1	−1	−1	−1	−1	−1	−1	−1	−1	19.8
6	+1	+1	−1	+1	+1	+1	−1	−1	−1	+1	−1	17.6
7	+1	−1	+1	+1	−1	+1	+1	+1	−1	−1	−1	16.7
8	+1	−1	−1	−1	+1	−1	+1	+1	−1	+1	+1	12.3
9	+1	+1	+1	−1	−1	−1	+1	−1	+1	+1	−1	14.5
10	−1	+1	−1	+1	+1	−1	+1	+1	+1	−1	−1	18.9
11	+1	+1	−1	−1	−1	+1	−1	+1	+1	−1	+1	14.9
12	−1	+1	+1	+1	−1	−1	−1	+1	−1	+1	+1	12.3

**Table 4:** Experimental variables for RSM at different levels used for production of Melanin

Factors	Variables	Units	Experimental values		
			−1	0	+1
A	Temperature	°C	40	45	50
B	Sodium dihydrogen phosphate	g	10	12	14
C	Moisture	%	10	17.5	25

### Extraction of melanin from *A. fumigatus* AFGRD105

Conidia were collected from *A. fumigatus* AFGRD105, grown for 5 days on SDA slants or plates, by adding 5 mL of sterile Phosphate Buffered Saline (PBS) of 1X concentration (8 g NaCl, 0.2 g KCl, 1.44 g Na_2_HPO_4_, 0.24 g of KH_2_PO_4_, pH 7.4, Distilled Water 1000 mL) and centrifuged at 8000 g for 30 min followed by washing in PBS thrice. A final wash was done using 1 M Sorbitol and 0.1 M Sodium Citrate (pH 5.5). 5 μL Macerozyme (10 mg/mL), as the cell lysing enzyme (Himedia; from *Rhizopus spp*), was added and incubated overnight at 30 °C to generate protoplasts. The protoplasts were collected by centrifugation and washed thrice by PBS and left overnight in 4.0 M Guanidine Thiocyanate (Himedia) at room temperature. The dark particles collected by centrifugation at 5000 g for 10 min were subjected to three washes using PBS followed by treatment using Reaction Buffer (10.0 mM Tris, 1.0 mM CaCl_2_ and 0.5 % SDS, pH 7.8) with 10 μL of 10 mg/mL Proteinase K and incubated at 37 °C. The debris obtained were boiled in 6.0 M HCl for 90 min.After treatment by boiling in acid, the melanin particles were collected by filtration through Whatmann paper and washed extensively with distilled water at 2 h intervals until a neutral pH was obtained. The pH of distilled water that is used to wash the crude melanin was checked using methyl orange for the pH comes close to seven indicating complete removal of the acid and lyophilized as required.

### Analysis of melanin and its biomass trend

The lyophilized particles was checked for colour; solubility in inorganic solvents (distilled water (pH 7), 1 N NaOH and 1 N HCl); solubility in organic solvents (ethanol, warm chloroform, warm acetone, benzene and phenol); precipitation (1 % ferric chloride, 1 N HCl and 1 N H_2_SO_4_); oxidation (6 % sodium hypochlorite, 30 % H_2_O_2_); and reduction (H_2_S and 5 % sodium hydrosulphite). Tests were carried out in parallel with Synthetic Melanin (Myko Teck Pvt Ltd, Goa) for comparison. For further validation of the results obtained using RSM, a comparative analysis was carried out on melanin production before and after optimization.

## Results

*Aspergillus fumigatus* has been exploited as a major source of secondary metabolites with potential commercial applications in the field of enzymes, pharmaceuticals, cosmetics and agriculture. This current study on optimization of melanin production plays a vital role in the cost effectiveness of melanin production.

**Fig. 1:**
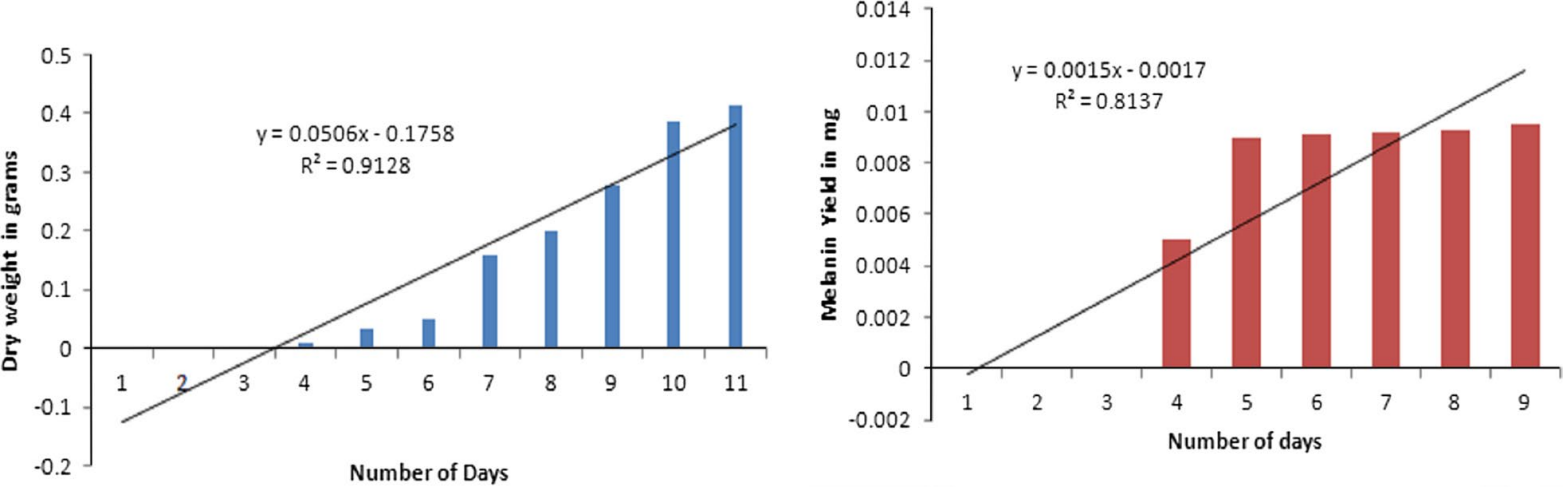
Variation in the production of dry weight and melanin production of the *A. fumigatus* AFGRD105 over a period of 10 days

The direct dry weight measurement of the *A. fumigatus* AFGRD105 grown on supplemented media resulted in typical sigmoid pattern. It was therefore observed in the present study that the dry matter weight of the substrate gradually decreased as the growth progressed in the case of *A. fumigatus* AFGRD105. The optimized time for growth was set at 5 days for optimizing the carbon and nitrogen sources as the melanin production is the same after day 5 and the conidial samples tend to become a dry mass of spores (Fig. 1).

**Fig. 2:**
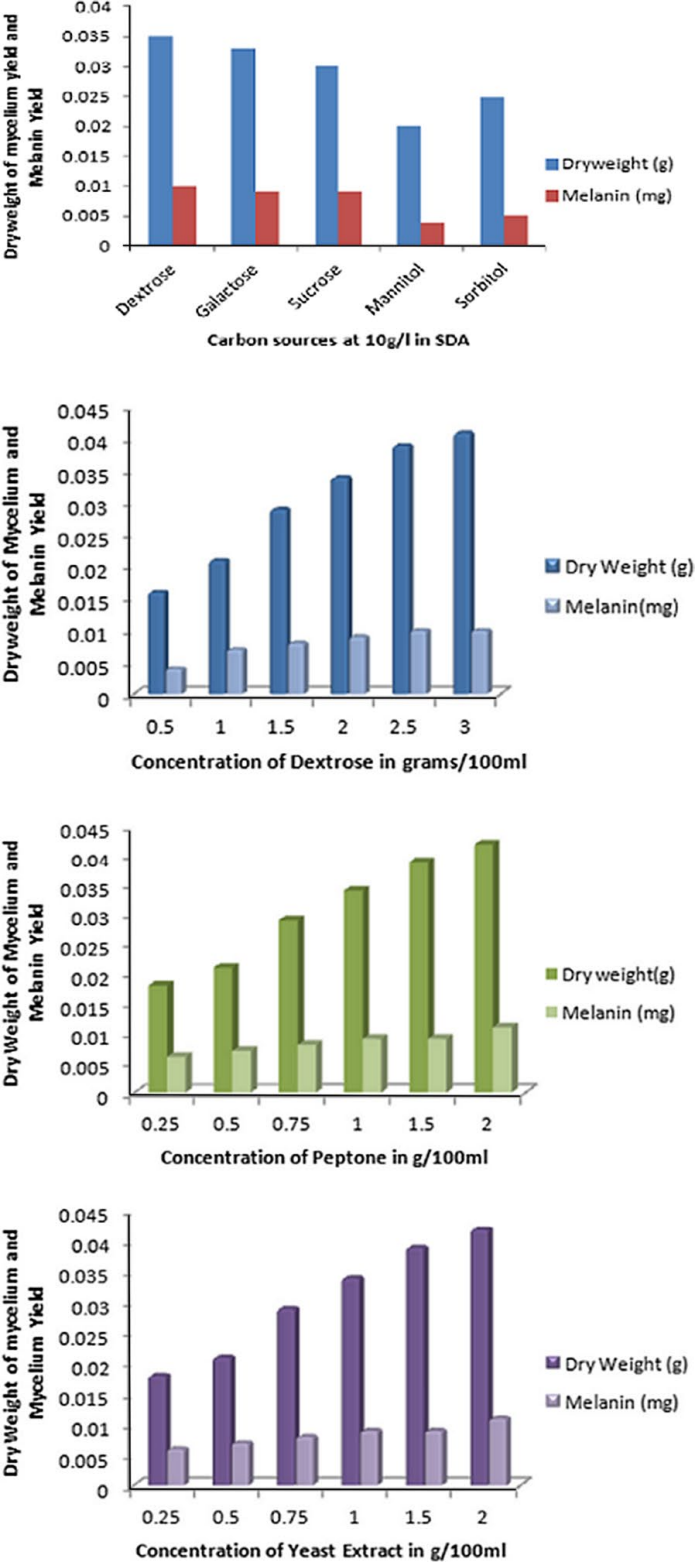
Effect of carbon source, dextrose, peptone and yeast extract on mycelial dry weight and melanin production by *A. fumigatus* AFGRD105

Of the carbon sources tested, it is found that Dextrose, which is the standard sugar used in Sabourauds Dextrose Agar medium, is the best carbon source for the growth of *A. fumigatus* AFGRD105 and also for melanin production. The other sources glucose, sucrose, and sorbitol show a descending increase in the dry weight of the fungus on the 5th day with mannitol being the lowest. The same feature is also observed for melanin production although the melanin production found least with mannitol can be attributed to the low dry weight of the mycelium. A gradient increase was however found as the amount of dextrose in the medium was increased (Fig. 2).

The effect of nitrogen source on both mycelial growth and melanin production was observed with the highest mycelial biomass achieved in the medium containing 1.0 g/100 mL of peptone for *A. fumigatus* AFGRD105. It is interesting to note that higher concentration of the nitrogen source is essentially not needed for mycelial growth which is opposite to the results for the carbon source (Fig. 2). Therefore, *A. fumigatus* AFGRD105 demonstrated enhanced production of mycelial biomass and melanin when cultured in media containing the unsubstituted SDA.

**Fig. 3:**
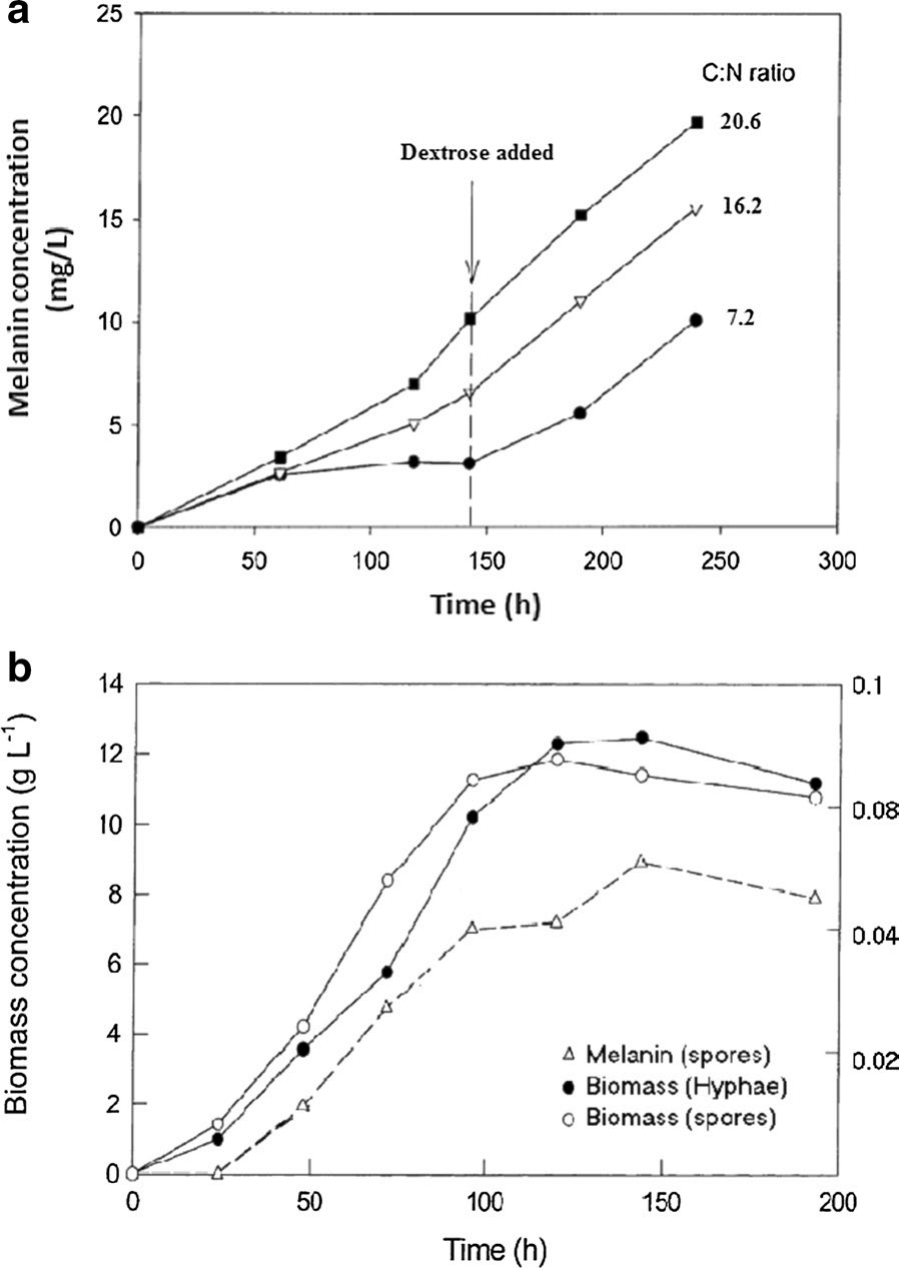
**a** Production of melanin for various C:N ratio. **b** Comparison of melanin production and biomass of *Aspergillus fumigatus* inoculated as hyphal and spore suspensions

For the initial values of C:N ratios of 7.2, 16.2 and 20.6, the amount of peptone as nitrogen source was held at the same level (Table 1) whereas the concentration of dextrose varied; thus the limiting source int his experiment was the nitrogen source. The production profile of melanin was showed in Fig. 3 confirming that the production of melanin in *Aspergillus fumigatus* is sources from spores unlike other metabolites that can be sourced from both hyphae and conidia. Addition of dextrose on day 6 was undertaken to maintain the final equivalent ratio of carbon and nitrogen sources added as 25.25 at all cases (Table 1). Figure 3 suggests that addition of dextrose did not affect the production of melanin due to limitation of nitrogen source but the presence excess dextrose from day 6 enhanced the rate of production of melanin.

The levels of the variables for the PB design were selected (Table 3) according to the previous single-factor experiments. Based on the selection, a 12-run PB experiment was chosen to pick up the main factors in the fermentative process for the production of melanin. Pareto chart showed that the values of temperature, moisture and Sodium dihydrogen phosphate was above the Bonferroni limit indicating significance of these factors. The main factors were picked up at the confidence level of 95 % based on their effects. According to the *t* test results, temperature, moisture and sodium dihydrogen phosphate were considered as the three major factors affecting the production. The rest of the factors level was below 90 % therefore considered insignificant. Experimental runs and their respective melanin yields are presented in Table 3. The adequacy of the model was checked using analysis of variance (ANOVA) which was tested using Fisher’s statistical analysis. The model F value of 5.05 implied that the model was significant and also showed that there was 0.25 % chance that the model F value could occur due to noise. The p values denotes the significance of the coefficients and also important in understanding the pattern of the mutual interactions between the variables.

The major factors including temperature, moisture and sodium dihydrogen phosphate were selected for further optimization by using BB. Based on the results of PB experiment, the BB experiment was designed and conducted, as is shown in Tables 3 and 5. Each of the three major factors (temperature, moisture and sodium dihydrogen phosphate) was designed in three levels (Table 4). The BB experiment results were submitted to ANOVA using the Design Expert software (version 8.0, Stat-Ease Inc., Minneapolis, USA), and the regression model was given as mentioned below which indicated that the experimental results of BB could be fitted into the final equation of factors as second order regression.

$$\begin{aligned} \bf{R1 }\left( {\textbf{Yield}} \right) & = \bf{0}\bf{.055135} -\bf{0.00359*A} + \bf{0.002987*B} + \bf{0.001471*C} - \bf{0.93*AB} \\ & \quad - \bf{0.0019*AC} - \bf{0.00011*BC} + \bf{0.0041A}^{\bf{2}} - \bf{0.0025B}^{\bf{2}} -\bf{0.0035C}^{\bf{2}} \\ \end{aligned}$$

where R1 is the yield of melanin obtained and A, B and C are the coded values for temperature, moisture and Sodium dihydrogen phosphate respectively.

**Table 5:** Seventeen trial Box–Behnken design matrix for the experimental variables with coded values

Runs	Experimental parameter			Response (mg/L)
	A	B	C	
1	−1	0	−1	16.5
2	+1	0	−1	17.1
3	−1	+1	0	13.5
4	0	−1	+1	23.5
5	0	0	0	19.5
6	0	0	0	14.3
7	+1	+1	0	13.2
8	+1	−1	0	20.1
9	−1	−1	0	19.5
10	0	0	0	11.7
11	0	0	0	12.3
12	−1	0	+1	22.5
13	0	−1	−1	29.3
14	+1	0	+1	31.9
15	0	+1	−1	22.4
16	0	0	0	21.6
17	0	+1	+1	32.6

The ANOVA of the quadratic regression model demonstrated the above mentioned equation is highly significant. It was in reasonable agreement with the predicted R^2^ of 0.8212. The lack-of-fit value for regression was not significant (0.1010), indicating that the model equation was adequate for predicting the melanin production under any combination of values of the variables.

**Fig. 4:**
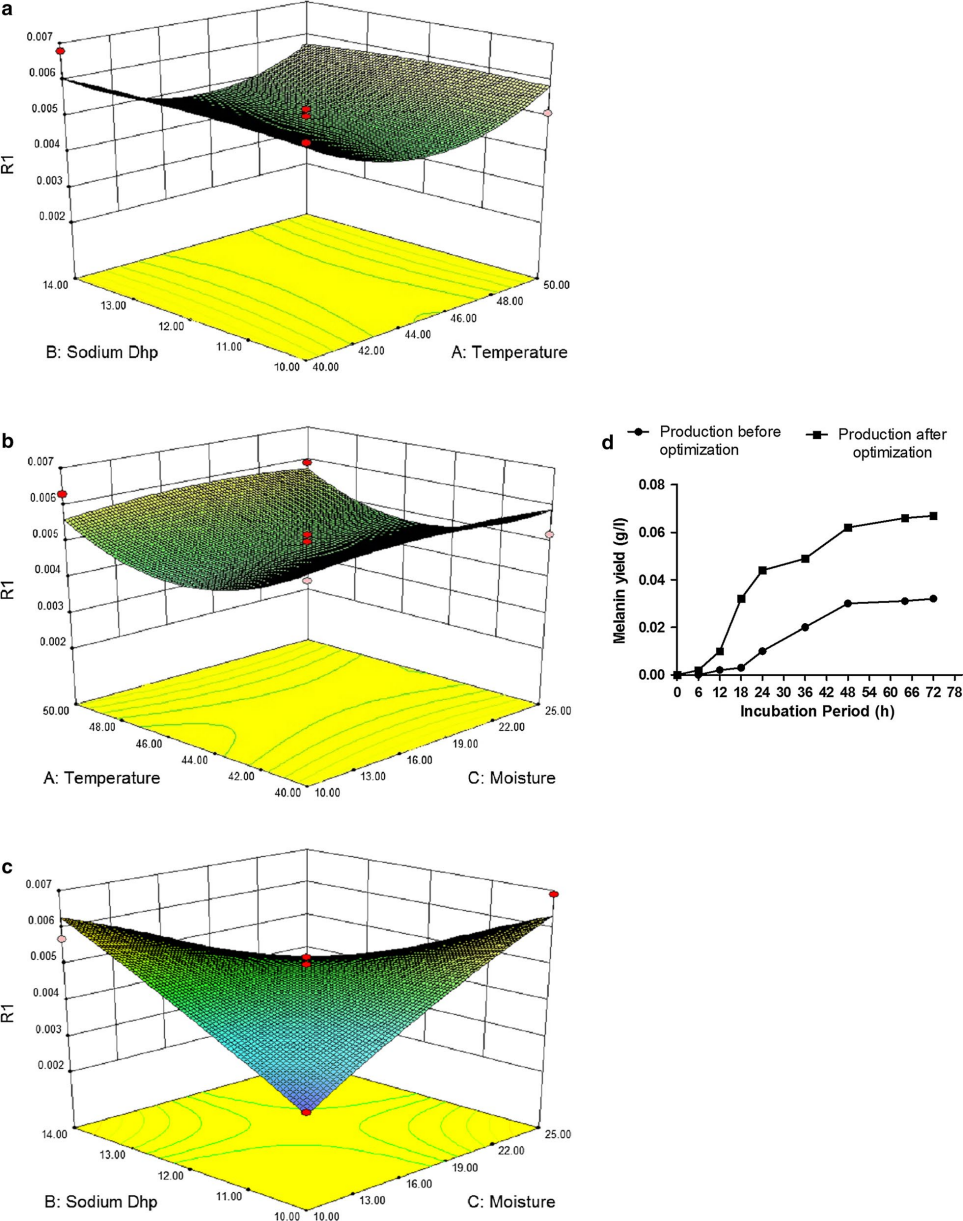
The surface *plots* of response surface methodology showing the effect of temperature, moisture and sodium dihydrogen phosphate and their mutual interaction on melanin production. **a** Sodium diphosphate and temparature; **b** Temperature and Moisture; **c** Moisture and Sodium diphosphate. **d** Melanin production before and after optimization

The present analysis carried out statistically using PB design showed the critical parameters that affected melanin production followed by further optimization using BB design determined the production was predominantly influenced by the amount of temperature, moisture and Sodium dihydrogen phosphate. The graphical representation provides a method to visualize the relationship between the response and experimental levels of each variable and the type of interactions between test variables in order to deduce the optimum conditions. The contour plots show the region of the desirability for the production of protein content with the point prediction from the analysis of variable for response surface cubic model for the production of melanin (Fig. 4).

The interaction effects and optimal levels of the variables were determined by plotting the three-dimensional (3D) response surface curves. The most efficient and economical conditions were to use the lowest concentrations of all the parameters for an optimal response. By using the response surface methodology, an attempt was made to understand the important variables to obtain an efficient response for maximum yield of melanin.

The pigment could not be dissolved in water, acid, ethanol, warm chloroform, warm acetone or benzene. The pigment was soluble in a concentrated alkaline solution or in phenol. The dissolved, extracted black pigment was lightened by the oxidizing agents NaClO and H_2_O_2_, as well as by the reducing agents H_2_S and Na_2_S_2_O_4_ (sodium hydrosulfite). The black pigment also reacted positively in a test for polyphenols with FeCl_3_, producing a flocculent brown precipitate. Our extracts reacted identically to synthetic melanin.

Melanin production of before and after optimization illustrated in Fig. 4d confirms the process parameters reached a higher concentration than the use of regular laboratory conditions. These process parameters were considered after the fungus was allowed to form greenish-grey conidia confirming the presence of melanin essentially after 4th day on an hourly basis. Optimization of melanin indicated the increase in the production of melanin from 3.4 mg/L to 6.6 mg/L resulting in a two fold increase after optimization. The biomass trend also indicates that the production of melanin is constant after a period of 5 days similar to the results obtained in Fig. 1. This production of melanin by *A. fumigatus* AFGRD105 without any the addition of any precursors and with the use of minimum medium components indicates a cost effective alternative.

## Discussion

Temperature and moisture have largely been associated with the fungal populations in aiding their growth even under lesser influential conditions (Nielsen et al. 2004; McGinnis 2007); whereas phosphates have largely been targeted in melanin related studies as an element in every composition of media (Alviano et al. 1991). The potential positive correlation of factors like temperature, moisture and phosphates may also be attributed to the results obtained on the direct dependency of growth of fungus with melanin production.

The production of melanin with respect to C:N utilization in the medium by fungi is the first to be undertaken in this study. Although various other fungi have been subjected production of biotechnologically prominent metabolites, this study pertains and confirms the production of melanin by the conidia of *Aspergillus fumigatus* as no melanin was obtained from the hyphae. With respect to these results, it can be confirmed that the yield of melanin can be improved by carbon although the growth has been arrested by nitrogen sources.

Statistical designs are effective tools that can be used to account for the main as well as the interactive influences of fermentation parameters on the process performance. Among them, RSM is a collection of certain statistical techniques for designing experiments, building models, evaluating the effect of the factors and searching for optimal conditions for desirable responses. Therefore, during the past decades, RSM has been extensively applied in the optimization of medium composition, fermentation conditions and food manufacturing processes (Vazquez and Martin 1997; Park et al. 2005).

Statistical designs has aided in analysing the important factors with minimum labour and low time consumption and also proved to be useful in optimizing medium composition for melanin production from *A. fumigatus* AFGRD105. Optimising the media conditions revealed a positive correlation in higher yield was influenced by temperature, moisture and Sodium dihydrogen phosphate.

In this present study, the statistical methodology, combination of the PB design and the Box–Behnken design, is demonstrated to be effective and reliable in selecting the statistically significant factors and finding the optimal concentration of those factors in the fermentation medium for melanin production (Bajaj and Singhal 2009; Yong et al. 2011). The interaction effects and optimal levels of the variables were determined by plotting the three-dimensional (3D) response surface curves. The shape of the response surface curves showed strong positive interaction between these tested variables.

Melanin production has also been detailed in *Escherichia coli, Kleibsiella sp., Bacillus thuringiensis* and *B. cereus* (Lagunas-Munoz et al. 2006; Shrishailnath et al. 2010; Chen et al. 2004; Zhang et al. 2007). The significant variables were quite different from this study and it may be due the fungal source of melanin in this study. Studies undertaken using *Brevundimonas sp.* SGJ showed increased yield of melanin with pH 5.31, tryptone 1.440 g/L, l-tyrosine 1.872 g/L and CuSO_4_ 0.0366 g/L. On comparision with the present study, use of RSM resulted in a 3.05-fold increase in melanin production (Surwase et al. 2013).

In this work, the process parameters—temperature, moisture and sodium dihydrogen phosphate were selected and optimized to produce melanin. Design Expert from Stat-Ease was used to develop the design of the experiment and BB design in RSM was used to optimize the process condition. Thus it has been concluded that the point prediction from the analysis of variable for response surface cubic model is used as basic tool for the production of melanin from *A. fumigatus* AFGRD105. This enhanced production of melanin can be further used in platforms of research in cosmetics and dyes.

## Abbreviations

RSM: response surface methodology
DoE: design of experiments
PCR: polymerase chain reaction
ITS: internal transcribed sequences
PB: Plackett Burman
BB: Box Behnken
ANOVA: analysis of variance
PBS: phosphate buffered saline
SSF: solid state fermentation

## References

[CR1] Abdel-Nabi MA, Ismail AMS, Ahmed SA, Abel Fattah AF (1998). Production and immobilization of alkaline protease from *Bacillus mycoides*. Bioresour Technol.

[CR2] Alviano CS, Farbiarz SR, De Souza W, Angluster J, Travassos LR (1991). Characterization of *Fonsecaea pedrosoi* melanin. J Gen Microbiol.

[CR3] Bajaj IB, Singhal RS (2009). Enhanced production of poly (γ-glutamic acid) from *Bacillus**licheniformis* NCIM 2324. Bioresour Technol.

[CR4] Bajaj IB, Lele SS, Singhal RS (2009). A statistical approach to optimization of fermentative *Licheniformis* NCIM 2324 by using metabolic precursors. Appl Biochem Biotechnol.

[CR5] Chen Y, Deng Y, Wang J, Cai J, Ren G (2004). Characterization of melanin produced by a wild-type strain of *Bacillus thuringiensis*. J Gen Appl Microbiol.

[CR6] Lagunas-Munoz VH, Cabrera-Valladares N, Bolıvar F, Gosset G, Martınez A (2006). Optimum melanin production using recombinant *Escherichia coli*. J Appl Microbiol.

[CR7] McGinnis MR (2007). Indoor mould development and dispersal. Med Mycol.

[CR8] Nielsen KF, Holm G, Uttrup LP, Nielsen PA (2004). Mould growth on building materials under low water activities. Influence of humidity and temperature on fungal growth and secondary metabolism. Int Biodeter Biodegr.

[CR9] Nikel PI, Pettinari MJ, Mendez BS, Galvagno MA (2005). Statistical optimization of a culture medium for biomass and poly (3-hydroxybutyrate) production by a recombinant *Escherichia coli* strain using agroindustrial byproducts. Int Microbiol.

[CR10] Park PK, Cho DH, Kim EY, Chu KH (2005). Optimization of carotenoid production by *Rhodotorula glutinis* using statistical experimental design. World J Microbiol Biotechnol.

[CR11] Plackett RL, Burman JP (1946). The design of optimum multifactorial experiments. Biometrika.

[CR12] Sangkharak K, Prasertsan P (2007). Optimization of polyhydroxybutyrate production from a wild type and two mutant strains of *Rhodobacter sphaeroides* using statistical method. J Biotechnol.

[CR13] Shrishailnath S, Kulkarni G, Yaligara V, Kyoung L, Karegoudar T (2010). Purification and physiochemical characterization of melanin pigment from *Klebsiella sp*. GSK. J Microbiol Biotechnol.

[CR14] Surwase SN, Jadhav SB, Phugare SS, Jadhav JP (2013). Optimization of melanin production by Brevundimonas sp. SGJ using response surface methodology. 3 Biotech.

[CR15] Vazquez M, Martin AM (1997) Optimization of *Phaffia rhodozyma* continuous culture through response surface methodology. Biotechnology 57:314–320. doi:10.1002/(SICI)1097-0290(19980205)57:3%3C314:AID-BIT8%3E3.3.CO;2-V 10099208

[CR16] Yong X, Raza W, Yu G, Ran W, Shen Q, Yang X (2011). Optimization of the production of poly-γ-glutamic acid by *Bacillus amyloliquefaciens* C1 in solid-state fermentation using dairy manure compost and monosodium glutamate production residues as basic substrates. Bioresour Technol.

[CR17] Zhang J, Cai J, Deng Y, Chen Y, Ren G (2007). Characterization of melanin produced by a wild-type strain of *Bacillus cereus*. Front Biol China.

